# Robotic hepatectomy for benign liver disease: single-centre consecutive series and meta-analysis comparing minimally invasive and open approaches

**DOI:** 10.1007/s11701-026-03692-z

**Published:** 2026-07-22

**Authors:** Graziano Ceccarelli, Pasquale Avella, Luca Ballelli, Lorenzo Mariani, Paolo Bianco, Riccardo Scarponi, Germano Guerra, Salvatore Spiezia, Aldo Rocca

**Affiliations:** 1 Division of General and Minimally Invasive Surgery, Department of Surgery, San Giovanni Battista Hospital, Foligno, Italy; 2 Minimally Invasive and Robotic Surgery Unit, San Matteo Hospital, Spoleto, Italy; 3 Department of Medicine and Health Science “V. Tiberio”, University of Molise, Campobasso, Italy; 4 Hepatobiliary and Pancreatic Surgery Unit, Pineta Grande Hospital, Castel Volturno, Italy; 5 Department of Clinical Medicine and Surgery, University of Naples “Federico II”, Naples, Italy

**Keywords:** Robotic hepatectomy, Benign liver disease, Minimally invasive surgery, Learning curve, Meta-analysis

## Abstract

Benign hepatic disease carries a distinctive ethical imperative to minimize perioperative morbidity in patients who might otherwise live asymptomatic lives. Minimally invasive approaches reduce pain, hospitalization, and recovery time, but laparoscopic resection of anatomically challenging posterosuperior segments remains technically demanding. Robotic hepatectomy may overcome these limitations. This study evaluates the safety, feasibility, learning curve, and comparative effectiveness of robotic hepatectomy in benign disease. We retrospectively analyzed a consecutive single-surgeon series of 62 robotic hepatectomies for benign disease, including a learning-curve analysis, and performed a parallel meta-analysis of 7 comparative studies stratified by surgical approach and lesion complexity. Robotic resection demonstrated excellent safety (1.6% major complication rate, 0% 90-day mortality, 0% readmissions) and reproducibility. The IWATE score reliably stratified cases and predicted operative duration. In the literature, robotic and laparoscopic resection showed comparable operative time, blood loss, and length of stay, with only a modestly longer robotic operative time in isolated resections (SMD + 0.52, *p* = 0.0046). Compared with open surgery, the robotic approach achieved significantly lower intraoperative blood loss (SMD − 0.94, *p* < 0.0001) and a shorter hospital stay (SMD − 1.32, *p* = 0.0253), with comparable operative time. The blood-loss advantage over open surgery was confirmed in strictly isolated benign resections (SMD − 0.71, *p* = 0.0004; I² = 0%). Robotic hepatectomy is safe, reproducible, and demonstrates clear learning curve plateau in benign disease and a stepping-stone strategy for malignant liver diseases. Robotic resection reliably accesses posterosuperior segments with low conversion rates, making it an attractive minimally invasive alternative that extends laparoscopic reach. In benign disease, where morbidity minimization and rapid recovery are paramount, robotic surgery offers a compelling new option. Future prospective, multicenter, randomized studies are needed for cost-effectiveness and quality-of-life analysis.

**Visual Abstract:**

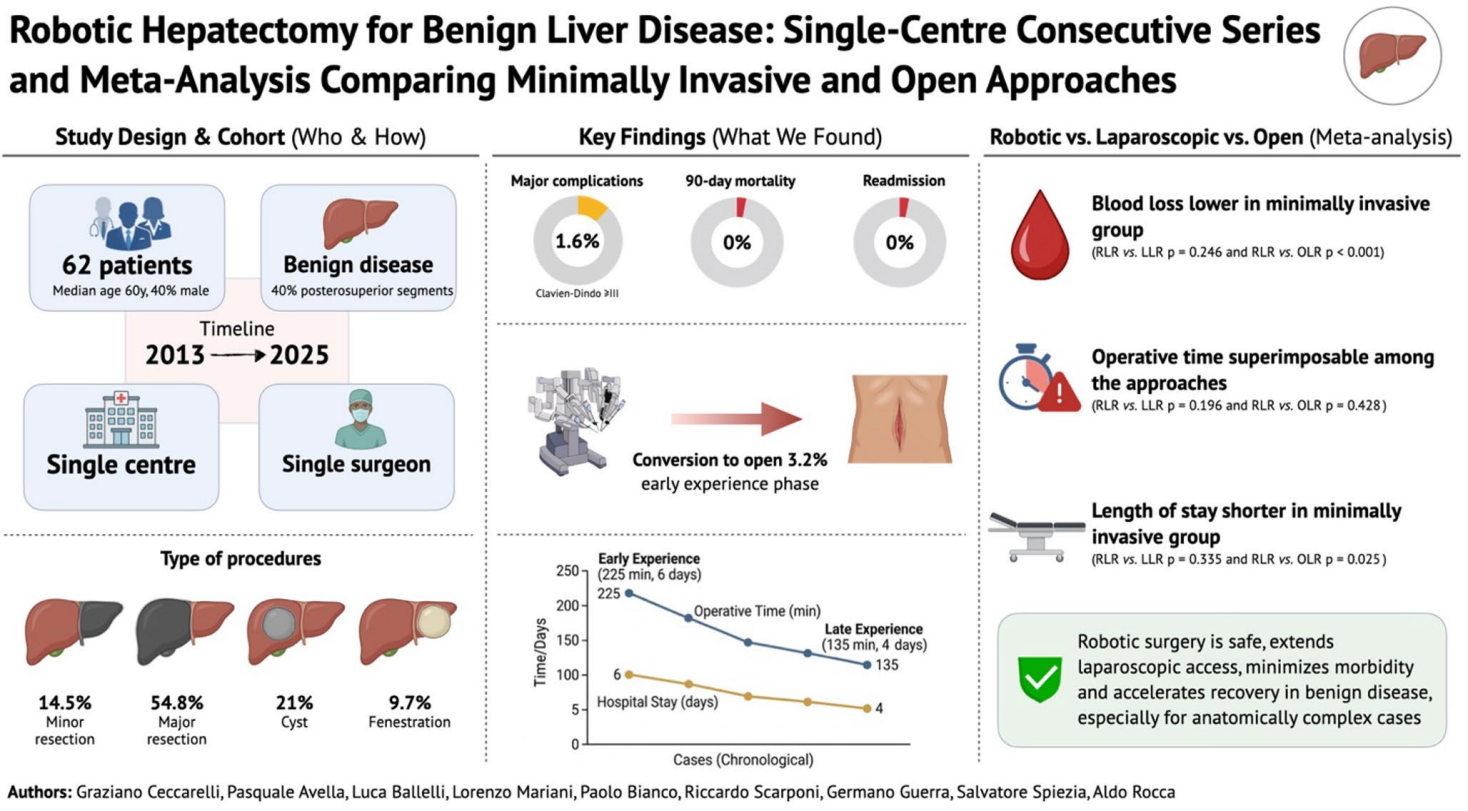

## Introduction

Benign hepatic lesions, encompassing cystic diseases, vascular tumours, hepatocellular adenomas, focal nodular hyperplasia, biliary cysts, and parasitic infections, are detected with increasing frequency on cross-sectional imaging performed for unrelated indications [1, 2]. While the majority can be managed conservatively, a meaningful subset requires surgical intervention for symptom relief, complication prevention, or diagnostic uncertainty [2].

Surgical management of benign liver disease poses a distinctive risk–benefit challenge: unlike oncological resections, where oncological adequacy may justify elevated procedural risk, operations for benign disease must carry the lowest achievable morbidity, emphasising parenchymal-sparing approaches and minimally invasive techniques [3, 4]. Since its introduction in the early 1990s, laparoscopic liver resection (LLR) has progressively become the standard for minor hepatectomies, demonstrating reduced blood loss, shorter hospitalisation, and faster functional recovery compared with open surgery [5, 6]. The Louisville [7], Southampton [8] and Brescia [9] consensus guidelines progressively extended LLR to major hepatectomies and technically demanding anatomical segments.

Robotic liver resection (RLR) has emerged as a technologically advanced evolution of minimally invasive hepatectomy [10, 11]. The robotic platform addresses intrinsic limitations of standard laparoscopy providing three-dimensional stereoscopic magnification, seven degrees of freedom, and motion scaling [12, 13]. These features confer advantages for resections in posterosuperior hepatic segments (I, IVa, VII, VIII) and for fine parenchymal dissection adjacent to major vascular structures [14, 15].

Despite growing evidence supporting RLR in oncological settings [16, 17], data specifically addressing robotic surgery for benign hepatic conditions remain scarce, largely confined to small case series or subgroup analyses within mixed cohorts [18].

Two specific gaps persist. First, no single-surgeon consecutive series exclusively dedicated to benign indications has been reported with adequate sample size. Second, although several comparative robotic–laparoscopic and robotic–open studies are now available, their quantitative synthesis through formal meta-analysis in the benign-disease context has not yet been performed. This knowledge gap is clinically relevant: the distinct patient profile, the primary emphasis on quality-of-life restoration and the need to justify the incremental technological cost of the robotic platform in a non-oncological setting represent unresolved issues.

The present study addresses these gaps by reporting the largest single-surgeon consecutive series of RLR exclusively for benign hepatic disease and characterising the learning curve and the relationship between resection complexity and perioperative outcomes.

Furthermore, we aim to evaluate the benchmark of the RLR in benign liver surgery providing a PRISMA-compliant systematic review with a focused random-effects meta-analysis of comparative studies.

## Materials and methods

### Study design and patient selection

A retrospective single-centre observational study of a prospectively maintained institutional database was conducted in accordance with the Strengthening the Reporting of Observational Studies in Epidemiology (STROBE) statement [19]. All consecutive adult patients (≥ 18 years) who underwent RLR at our institution from January 2013 to January 2025 were screened.

Inclusion criteria: primary benign hepatic disease confirmed on final histology, or hepatic resection performed concomitantly with oncological surgery where the hepatic specimen proved benign on definitive pathology. All types of hepatic surgery were eligible: cyst fenestration/deroofing, cyst excision/pericystectomy, anatomical and non-anatomical resection.

Exclusion criteria: confirmed malignant primary or metastatic hepatic disease on final histology. Concomitant resection of other organs was not an exclusion criterion.

Surgical indications were established in accordance with the 2022 Benign Liver Lesions Guidelines [20] and the 2016 EASL Guidelines on Benign Liver Tumours [21]. All patients provided written informed consent. The study was conducted in compliance with the Declaration of Helsinki.

The present series builds upon prior collaborative experience developed in preceding years between the Robotic Surgery Unit of Spoleto and the Hepato-Pancreato-Biliary (HPB) Referral Center of A.O.R.N. A. Cardarelli Hospital in Naples, under the direction of Prof. L. Casciola and Prof. F. Calise [22, 23].

Although no formal institutional agreement was established between the two centers, the surgical teams routinely engaged in collegial case-by-case discussion, and surgeons were granted dedicated authorizations to operate across both institutions, thereby facilitating the consolidation of their learning curves and providing intraoperative tutoring to colleagues during the most technically demanding procedures.

## Data collection

Clinical and operative data were retrieved from institutional electronic medical records and dedicated surgical databases and subsequently anonymized prior to analysis.

Collected variables encompassed patient demographic characteristics (age and sex), comorbidity profile, history of prior abdominal surgery, and physical status according to the American Society of Anesthesiologists (ASA) classification [24].

The extent of hepatic resection was classified following the Brescia Guidelines [9] as minor (fewer than three segments), major (three or more segments), or complex (involving the caudate lobe or segments 4a, 7, 8). Liver resection was defined as any anatomical hepatectomy or non-anatomical parenchymal resection performed with curative intent for complete lesion removal.

Intraoperative variables included the surgical approach, conversion to open or laparoscopic surgery, total operative time, use and duration of the Pringle maneuver (intermittent or continuous), estimated intraoperative blood loss, and performance of concomitant procedures.

Surgical difficulty was assessed using the IWATE difficulty score, a validated instrument applicable to both laparoscopic and robotic hepatectomy [25].

Postoperative outcomes included admission to the intensive care unit (ICU) and its duration, transfusion requirements, use of total parenteral nutrition, other supportive interventions, total length of hospital stay, and in-hospital mortality. Complications were graded according to the Clavien–Dindo classification [26] with major morbidity defined as grade III or above [27, 28].

For the present analysis, any liver failure or bile leakage necessitating invasive organ support (e.g., dialysis, mechanical ventilation) was considered grade IV (IVa = single-organ, IVb = multiple-organ failure). Death within 90 days of resection was classified as grade V.

Postoperative mortality was defined as any death within 90 days after surgery or before hospital discharge, whichever occurred later.

## Surgical technique

All procedures were performed by a single surgeon (G.C.) using the da Vinci Xi^®^ Surgical System (Intuitive Surgical, Sunnyvale, CA, USA); the da Vinci S^®^ was used for cases performed before 2017. Patients were positioned in supine decubitus with the lower limbs abducted, with the assistant between the legs. The operating table was tilted 10–15° in reverse Trendelenburg position and rotated according to liver segments.

Four robotic trocars were placed in configurations adapted to the target hepatic lobe (right vs. left), with one or two accessory trocars, at least one of which was equipped with the AirSeal^®^ system (CONMED, Utica, NY, USA). Laparoscopic adhesiolysis was performed before robotic docking when required, followed by intraoperative ultrasound (IOUS) for resection planning and identification of occult lesions [23, 29]. Parenchymal transection was performed using the Maryland bipolar forceps, Kelly-clamp technique, and laparoscopic accessory instruments including Aquamantys^®^ bipolar sealer (Medtronic, Minneapolis, MN, USA) through the accessory ports.

Indocyanine green (ICG) fluorescence (0.25 mg/kg intravenous bolus, 24–48 h preoperatively for intraoperative localisation of liver neoplasm; 0.05 mg/kg intraoperatively for biliary leak detection) was used routinely for parenchymal margin guidance and intraoperative biliary mapping [13]. The Pringle manoeuvre was systematically prepared using a vessel loop or a Foley catheter according to Huang’s loop [30] and applied selectively at the surgeon’s discretion, case by case. Operative time was recorded from skin incision to closure (including cases where other surgical procedures were performed), including robotic docking time (single docking for isolated hepatectomies; double docking for combined colorectal–hepatic procedures).

## Learning curve analysis

To assess the learning curve, the cohort was divided chronologically into an early experience group (first 31 cases) and a late experience group (last 31 cases). Primary perioperative outcomes were compared between groups using non-parametric tests. IWATE scores were compared between phases to control for case-selection bias, as previously validated in robotic learning curve analyses.

## Literature review

**Fig. 1:**
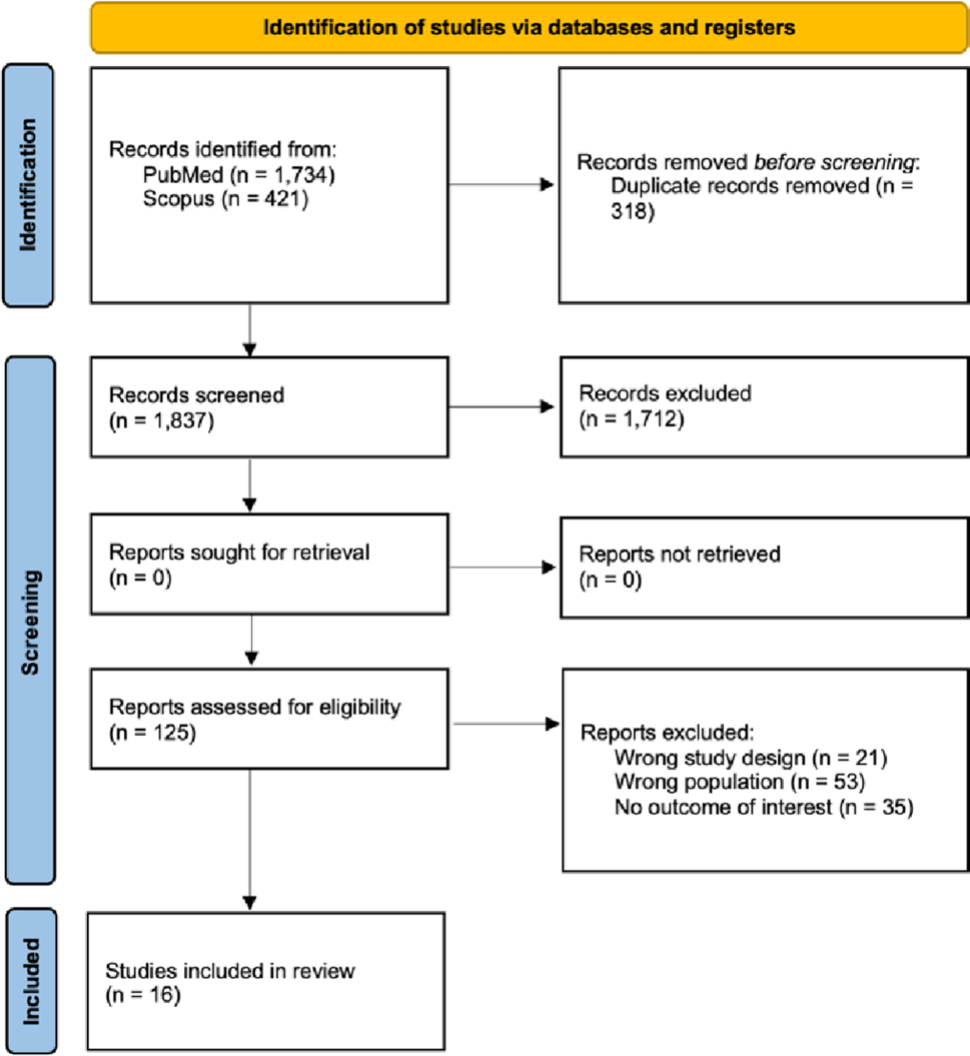
PRISMA flowchart

A systematic search was conducted in accordance with PRISMA 2020 guidelines across two electronic databases: PubMed/MEDLINE and Scopus (Fig. 1). The search was performed in January 2026 and updated on 29 April 2026. The following MeSH and free-text terms were applied in Boolean combination: (“robotic liver resection” OR “robotic hepatectomy” OR “robot-assisted liver surgery”) AND (“benign liver disease” OR “liver cyst” OR “hepatic haemangioma” OR “hepatocellular adenoma” OR “focal nodular hyperplasia” OR “hydatid cyst” OR “biliary cyst” OR “polycystic liver” OR “hepatic hamartoma”). No language or date restriction was applied at the search stage.

Inclusion criteria: (1) original studies (case series, cohort studies, comparative studies, propensity-score matched analyses) reporting outcomes of RLRs; (2) cohort including at least some benign indications or reporting subgroup data on benign disease; (3) reporting ≥ 1 of the following outcomes: operative time, conversion rate, intraoperative blood loss, length of hospital stay, morbidity/mortality.

Exclusion criteria: (1) case reports or studies with total number of patients < 5; (2) review articles, editorials, or letters; (3) animal or cadaveric studies; (4) studies exclusively addressing malignant disease without extractable benign subgroup data; (5) duplicate publications; (6) studies not providing sufficient data for quantitative extraction.

PROSPERO registration was not undertaken, as the systematic review and meta-analysis were conceived as an evidence-synthesis component complementary to the institutional cohort rather than as a stand-alone protocol; nevertheless, the search strategy, eligibility criteria, and analytical plan were defined a priori and reported in compliance with PRISMA 2020 guidelines.

### Statistical analysis

Continuous variables are expressed as median and interquartile range (IQR); categorical variables as absolute frequencies and percentages. Between-group comparisons used the Mann–Whitney U test for continuous variables and Fisher’s exact test for categorical variables. Spearman’s rank correlation was used to assess the association between IWATE score and perioperative outcomes. Cases with combined hepatic and non-hepatic surgery were excluded from the IWATE–operative time correlation analysis to remove operative time confounding. Statistical significance was set at *p* < 0.05 (two-tailed). All analyses were performed using SPSS version 28.0 (IBM Corporation, Armonk, NY, USA). No imputation was performed for missing data, as the prospectively maintained database yielded complete case records for all primary outcomes.

Comparative meta-analyses were conducted to assess operative time, intraoperative blood loss, and length of hospital stay across robotic, laparoscopic, and open hepatectomy approaches. Data extraction was stratified into two distinct cohorts: (1) all screened comparative studies, irrespective of operative scope, and (2) studies reporting isolated hepatic resection only (excluding no liver resection). Pooled standardised mean differences (SMD) with Hedges’ g and 95% confidence intervals (CI) were calculated under random-effects models. Heterogeneity was quantified using the I² statistic and tau-squared (τ²). Statistical analyses were performed using Comprehensive Meta-Analysis software version 4.0 (Biostat, Inc., Englewood, NJ, USA). Significance was established at α = 0.05 (two-tailed).

## Ethical considerations

The study was conducted in accordance with the Declaration of Helsinki. Approval was obtained from the Institutional Review Board of the coordinating center (University of Molise, protocol no. 25170, approved May 29, 2024).

## Results

### Patient demographics and clinical characteristics

**Fig. 2:**
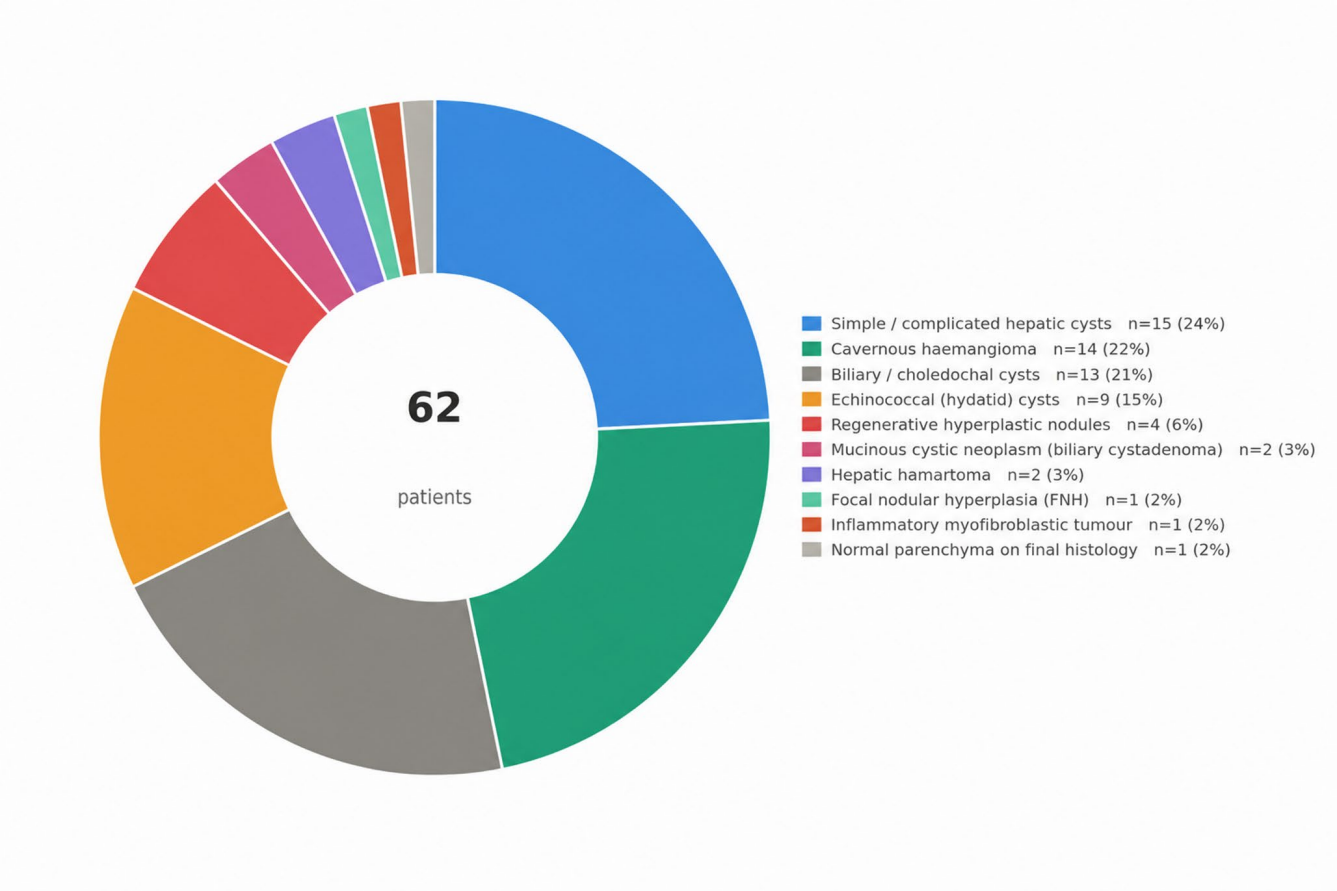
Distribution of benign hepatic lesions by histological diagnosis

The study included 62 consecutive patients (Male: Female = 25:37). Median age was 60 years (IQR 51–69; range 18–82) and median Body Mass Index (BMI) was 26.6 kg/m² (IQR 24.1–28.9; range 14–30). Comorbidities were present in 27 patients (43.5%), with a median ASA score of 2 (IQR 1–3; range 1–3). Patient demographic, clinical characteristics and resection details are reported in Table 1. Histological diagnoses are detailed in Fig. 2.

**Fig. 3:**
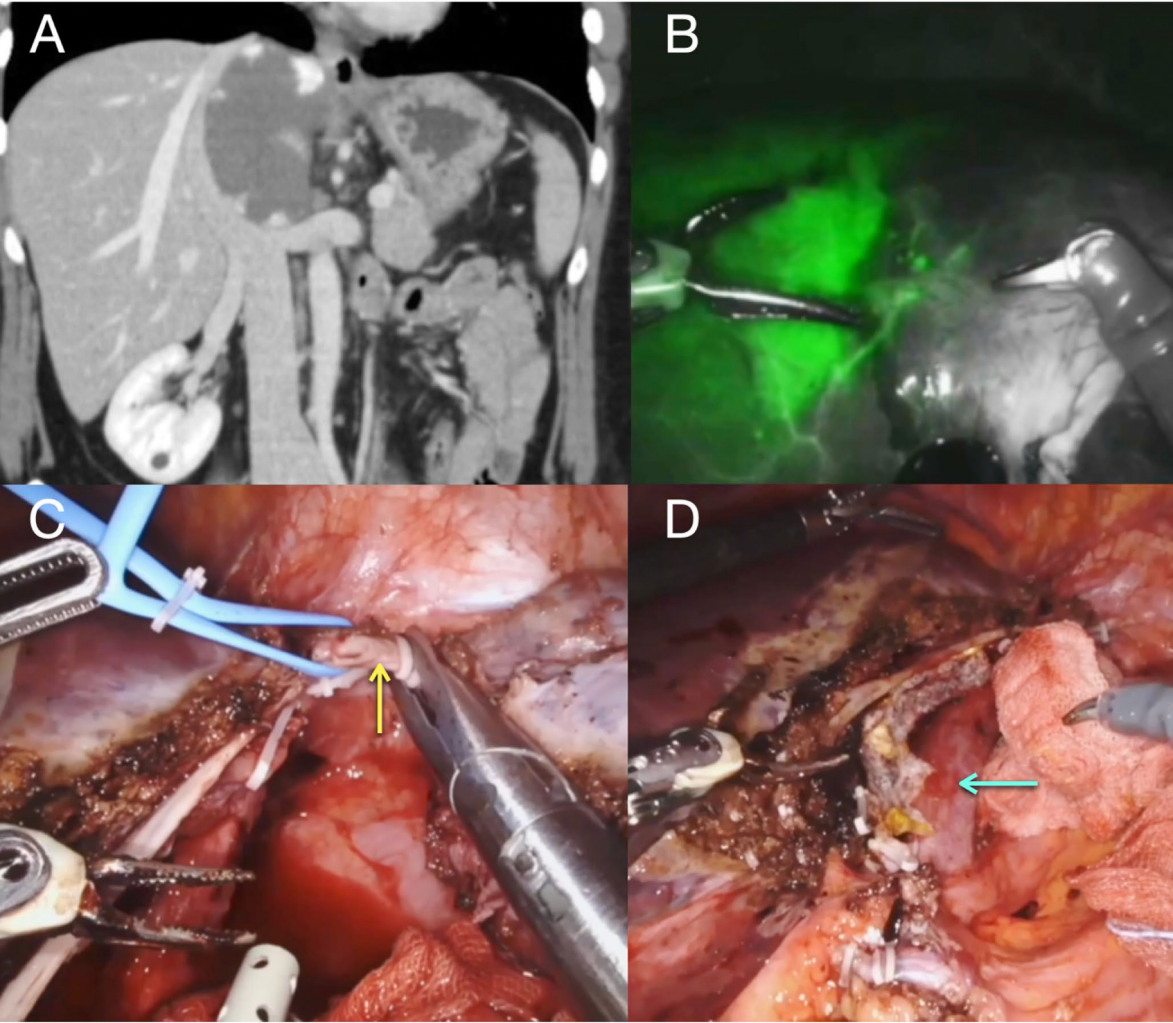
Robotic liver resection for a giant hepatic haemangioma compressing the inferior vena cava. **(A)** Hepatic haemangioma compressing the inferior vena cava and involving liver segments I, II, III, and IVa. **(B)** Indocyanine green (ICG) administration for negative-staining assessment following ligation of the Glissonean pedicles feeding the haemangioma. **(C)** Division of the left hepatic vein between Hem-o-lok clips (yellow arrow). **(D)** Hepatic transection surface with exposure of the inferior vena cava (blue arrow) at the completion of the procedure

Figure 3 shows CT and intraoperative findings of liver haemangioma.

**Table 1:** Patient demographic and surgical details

Variable	Value (*n* = 62)
Gender — Male / Female	25 / 37
Age, years — median (IQR)	60 (51–69)
BMI, kg/m² — median (IQR)	26.6 (24.1–28.9)
Comorbidities present, n. (%)	27 (43.5)
ASA score — median (IQR)	2 (1–3)
Follow-up period, months — median (IQR)	36 (18–72)
Robotic approach initiated, n. (%)	62 (100)
- Major hepatectomy (≥ 3 segments)	9 (14.5)
- Minor resection (≤ 2 segments)	34 (54.8)
- Cyst excision / pericystectomy	13 (21)
- Fenestration (deroofing)	6 (9.7)
- Concomitant cholecystectomy	13 (21)
Complex resections (I, IVa, VII, VIII), n. (%)	25 (40.3)
Combined hepatic + colorectal/other procedures, n. (%)	16 (25.8)
Conversion to open surgery, n. (%)	2 (3.2)
Conversion to laparoscopy, n. (%)	0 (0)
Operative time, min — median (IQR)	210 (180–260)
Estimated blood loss, mL — median (range)	150 (20–300)
Pringle manoeuvre, n. (%)	7 (11.3)
Pringle duration, min — median (IQR)	28 (20–35)
ICG fluorescence, n. (%)	55 (88.7)
Intraoperative ultrasound (IOUS), n (%)	62 (100)
Hospital stay, days — median (IQR)	6 (1–8)
Overall complication rate (Clavien–Dindo ≥ I), n (%)	7 (11.3)
Clavien–Dindo ≥ III, n (%)	1 (1.6)
- Grade IIIa (radiological intervention)	1 (50)
90-day mortality	0 (0)
90-day readmission	0 (0)

### Learning curve analysis

Comparison between the early (*n* = 31) and late (*n* = 31) experience groups is presented in Table 2.

**Table 2:** Learning curve analysis: early vs. late experience (*n* = 31 vs. *n* = 31 patients)

Variable	Early (*n* = 31)	Late (*n* = 31)	*p*-value
Operative time, min — median (IQR)	225 (172.5–310)	135 (112.5–225)	**0.033**
Estimated blood loss, mL — median (IQR)	300 (120–300)	45 (20–100)	0.210
Pringle manoeuvre use, n (%)	5 (16.1)	4 (12.9)	0.999
Pringle duration, min — median (IQR)	22.5 (18.3–25.8)	35 (31.5–42.5)	0.074
Conversion to open, n (%)	2 (6.5)	0 (0)	0.490
Hospital stay, days — median (IQR)	6 (5–8)	4 (1–5.5)	**0.004**
Clavien-Dindo ≥ III complications, n (%)	1 (3.2)	0 (0)	0.999
IWATE score — median (IQR)	4 (2–5)	5 (3–7)	0.299

Operative time decreased significantly from a median of 225 min (IQR 172.5–310) to 135 min (IQR 112.5–225) (*p* = 0.033). Hospital stay was significantly reduced (6 vs. 4 days; *p* = 0.004). Intraoperative blood loss showed a clinically meaningful decline (300 vs. 45 mL) that did not reach statistical significance (*p* = 0.21), likely reflecting the limited statistical power for this continuous variable in the available sample. The rate of clinically relevant bleeding, Pringle manoeuvre use, and major complications did not differ significantly between phases (*p* = 0.999). Both conversions occurred in the early experience group (6.5% vs. 0%; *p* = 0.49). Pringle duration showed a non-significant trend toward increase in the late phase (35 vs. 22.5 min; *p* = 0.074), consistent with selective ischaemic management of more complex cases in the advanced experience period.

IWATE scores were comparable between early and late phases (*p* = 0.299), confirming absence of case-selection bias. Mean IWATE score was 4.9 ± 2.6 (median 4, IQR 3–7), indicating a predominance of low-to-intermediate complexity procedures. No significant difference in IWATE score was found between early and late phases (Table 2), confirming comparability of case complexity across the learning curve. In the overall cohort (including combined procedures), Spearman correlation between IWATE score and operative time was non-significant (ρ = 0.21; *p* = 0.18), reflecting confounding from the additional operative time required by concomitant non-hepatic procedures. After exclusion of combined colorectal–hepatic cases, IWATE score correlated significantly with operative time in isolated hepatectomies (Spearman ρ = 0.60; *p* = 0.0004), confirming its validity as a complexity predictor in the robotic setting. IWATE score did not correlate significantly with blood loss or hospital stay (Table 3).

**Table 3:** Spearman rank correlations: IWATE Score vs. perioperative outcomes

Outcome	Spearman ρ	*p*-value
Operative time (all cases, *n* = 62)	0.21	0.180
Operative time (isolated hepatectomies, *n* = 46)*	0.60	**0.0004**
Estimated blood loss	0.17	0.550
Hospital stay	−0.01	0.960

*Isolated hepatectomies analysis was performed excluding combined colorectal and liver procedures

### Robotic vs. laparoscopic surgery: literature experiences

The PRISMA 2020 flow is summarised as follows: 2,155 records were identified from databases; after removal of 318 duplicates, 1,837 records were screened by title and abstract. Of these, 1,712 were excluded as not meeting inclusion criteria. Of 125 full-text articles assessed for eligibility, 16 studies were ultimately included in the qualitative synthesis, while 7 were included in the meta-analysis. (Tables 4 and 5). Most frequent indications were cystic disease, haemangiomas, focal nodular hyperplasia, and hepatolithiasis.

**Table 4:** Baseline characteristics and perioperative outcomes of patients underwent robotic surgery for benign liver diseases

Author	Year	Country	Study Design / Years	BLD Patients / Total	Males (*n*)	BMI (kg/m²)	Age (years), Mean ± SD or Median (range)	Type of Procedure	Pathology	Operative Time (min)	Blood Loss (mL)	Blood Transfusion	Conversion Rate	Length of Stay (days)	Perioperative Mortality	Complications
Kandil et al. [31]	2013	USA	Retrospective; Feb–Aug 2011	3/7	2	(1) 26.4 / (2) 36.5 / (3) 28.1	(1) 21 / (2) 45 / (3) 64	LLS; LH; Single-port LLS	Hepatic adenoma 1; FNH 1; Adenoma 1	(1) 90 / (2) 79 / (3) 51	(1) 200 / (2) 200 / (3) 15	0 (0%)	0 (0%)	(1) 1 / (2) 2 / (3) 5	0 (0%)	Delirium/tremors in patient #3
Nota et al. [32]	2015	The Netherlands	Technical report	2/2	0	NA	(1) 32 / (2) 51	Cyst fenestration 2	Hepatic cyst 1; Polycystic liver disease 1	(1) 90 / (2) 115	(1) 10 / (2) 20	NA	NA	(1) 1 / (2) 3	0 (0%)	0 (0%)
Lee et al. [33]	2016	China	Retrospective; Sep 2010–Apr 2015	15/15	3	NA	56.5 (35–79)	LLS 10; LH 3; RH 2	Hepatolithiasis 15 (100%)	313.9 (137–620)	236 (10–700)	2 (13.3%)	0 (0%)	9.7 (4–22)	0 (0%)	4 (26.7%)
Kim et al. [34]	2016	South Korea	Retrospective; May 2007–Jul 2013	4/12	1	NA	62.3 ± 6.6	LLS 4	Hepatolithiasis 4 (80%); Hepatic cyst 1 (20%)	455.8 ± 144.2	250 (123–462) median (IQR)	NA	0 (0%)	7 (7–7.5) median (IQR)	0 (0%)	1
Goja et al. [35]	2017	India	Case series; Feb 2015–Jan 2016	4/10	3	NA	(1) 15 / (2) 44 / (3) 55 / (4) 59	Deroofing cysts 2; Partial cystectomy 2; LH 2	Polycystic liver disease 2; Hemangioma 1; CE 1	527.5 (370–660)	NA	1 (25%)	0 (0%)	11.3 (4–28)	0 (0%)	1 (25%)
Magistri et al. [36]	2019	Italy	Retrospective	15/15	8	23.05 (21–29)	51 (24–76)	RH 1; LLS 2; Segmentectomy 5 (1 caudate); Wedge resection 3; Cyst-pericystectomy 5	CE 16 (100%)	210 (95–550)	100 (50–550)	NA	0 (0%)	4 (3–13)	0 (0%)	4 (26.7%)
Tsirlis et al. [37]	2019	UK	Retrospective; Jan 2016–Jan 2018	17/17	2	NA	64 (42–83)	Robotic fenestration/deroofing 17; Cholecystectomy 5	Hepatic cyst (100%)	174 (97–335)	16 cases < 50; 1 case 200	NA	0 (0%)	2.5 (1–10)	0 (0%)	2 (11.8%)
Shu et al. [38]	2019	China	Retrospective PSM; Oct 2010–Aug 2017	26/26	9	NA	53 (20–70)	LLH 3; LH 16; RH 4; Segmentectomy 2; Hilar plasty 1; +CBD reconstruction 6; +CBD exploration 12	Hepatolithiasis 26 (100%)	376.69 ± 129.05	315.38 ± 237.81	4 (15.4%)	1 (3.8%)	13.54 ± 6.54	0 (0%)	12 (46.2%)
Lee et al. [39]	2019	South Korea	Retrospective; Jun 2016–Apr 2018	2/13	1	24.6 ± 4.2	(1) 69 / (2) 69	LH 2	Biliary cyst 1; Hepatolithiasis 1	(1) 195 / (2) 220	NA	0 (0%)	0 (0%)	(1) 7 / (2) 14	0 (0%)	Fluid collection in patient #2
Giulianotti et al. [40]	2019	USA	Retrospective; Jul 2015–Nov 2018	3/3	3	(1) 24.87 / (2) 27.36 / (3) 25.49	(1) 42 / (2) 45 / (3) 61	Robotic enucleation of angioma 3; Cholecystectomy 1	Hemangioma 3 (100%)	(1) 146 / (2) 121 / (3) 193	(1) 100 / (2) 50 / (3) 600	1 (33.3%)	0 (0%)	(1) 3 / (2) 4 / (3) 5	0 (0%)	Pulmonary embolism in patient #3
Hu et al. [41]	2020	China	Retrospective; Apr 2011–Apr 2017	19/19	2	NA	49.2 ± 10.6	Hemihepatectomy 19 (100%)	Hemangioma (100%)	256.3 ± 57.7	319.5 ± 206.0	5 (26.3%)	NA	5.5 ± 2.1	0 (0%)	1 (5.3%)
Zhao et al. [42]	2020	China	Retrospective; Sep–Dec 2019	5/5	5	25.68 (21.22–30.10)	38.6 ± 12.4 (24–56)	Caudate lobectomy 2; Hepatectomy seg. VII 1; Pericystectomy seg. VIII 1; RH 1	CE 4 (80%); AE 1 (20%)	242 (175–300)	210 (50–600)	1 (20%)	0 (0%)	10.8 (5–19)	0 (0%)	5 (100%)
Steinkraus et al. [43]	2022	Germany	Case series; Jan 2021-Aug 2022	16/16	4	25.8 (20.2–36.8)	55.5 (23–73)	Minor resection (≤ 3 segments) 12; Major resection (> 3 segment) 4	AE 14	251 (160–395)	300 (100–1500)	2 (12.5%)	0 (0%)	7 (4–30)	0 (0%)	Clavien-Dindo I-II 3; Clavien-Dindo ≥ III 1
Zhang et al. [44]	2022	China	Case series; Oct 2019-Jun 2021	7/7	3	19 ± 4.24	43.1 ± 11.6	Pericystectomy 7	CE 7	225.43 ± 44.75	214.29 ± 265.70	NA	0 (0%)	7.29 ± 4.31	0 (0%)	Abdominal effusion 2; Pulmunary infection 1
Zhang et al. [45]	2023	China	Retrospective PSM; Feb 2015-Jun 2021	43/43	13	22.4 (18.8–32.9)	48 (26–62)	Enucleation involving 1 or 2 segments 17; LLS 16, Enucleation involving ≥ 3 segments 6; LH 4	Hemangioma 43	270 (120–480)	200 (50 -1500)	3 (7%)	2 (4.7%)	7 (4–13)	0 (0%)	Bile leak 1; Pleural effusion 4
Wu et al. [46]	2025	China	Retrospective*; Jan 2020-Jun 2024	12/12	4	NA	93.1 ± 33 months	NA	FNH 12	160 (140–180)	20 (16.2–27.5)	NA	NA	4.5 (4–7)	0 (0%)	5 (41.66%)

Abbreviations: BLD, Benign Liver Disease; BMI, Body Mass Index; CE, Cystic Echinococcosis; AE, Alveolar Echinococcosis; FNH, Focal Nodular Hyperplasia; LLS, Left Lateral Sectionectomy; LH, Left Hepatectomy; RH, Right Hepatectomy; CBD, Common Bile Duct; NA, Not Available; PSM, Propensity Score Matching.*Paediatric cohort

**Table 5:** Baseline characteristics and perioperative outcomes of patients underwent robotic surgery for benign liver diseases

		Operative time (min), mean ± SD or median (range)	Blood loss (mL), mean ± SD or median (range)	Blood transfusion rate, n. (%)	Length of stay (days), mean ± SD or median (range)	Conversion to open surgery, n. (%)	Complications, n. (%)	Mortality, n. (%)	Cost (USD), mean ± SD or median (range)
Lee et al. [33]	**RLR (** *n* ** = 10)**	214 (137–280)	50 (10–500)	0 (0)	5.5 (4–7)	0 (0)	0 (0)	0 (0)	3,200*
	**OLR (** *n* ** = 27)**	220 (115–405)	200 (40–684)	1 (3.7)	8 (5–22)	—	9 (33.3)	0 (0)	1,350*
	***P value***	*0.906*	***0.001***	*0.999*	***0.001***	*—*	*0.079*	*0.999*	*NA*
Kim et al. [34]	**RLR (** *n* ** = 4)**	455.8 ± 144.2	250 (123–462)	NA	7 (7–7.5)	0 (0)	0 (0)	0 (0)	8,017*
	**LLR (** *n* ** = 5)**	330.6 ± 123.5	350 (150–500)	NA	6 (6–7)	0 (0)	1 (20)	0 (0)	7,437*
	***P value***	*0.203*	*0.762*	*NA*	*0.317*	*0.999*	*0.999*	*0.999*	*NA*
Shu et al. [38]	**RLLS (** *n* ** = 26)**	376.69 ± 129.05	315.38 ± 237.81	4 (15.4)	13.54 ± 6.54	1 (3.8)	12 (46.2)	0 (0)	15,239 ± 4,499
	**OLR (** *n* ** = 52)**	319.15 ± 127.58	542.88 ± 518.70	24 (46.2)	17.81 ± 7.49	—	33 (63.5)	2 (3.8)	12,173 ± 5,372
	***P value***	*0.065*	***0.037***	***0.008***	***0.016***	—	*0.196*	*0.549*	***0.014***
Hu et al. [41]	**RLR (** *n* ** = 19)**	256.3 ± 57.7	319.5 ± 206.0	5 (26.3)	5.5 ± 2.1	NA	1 (5.3)	0 (0)	NA
	**LLR (** *n* ** = 13)**	268.4 ± 93.6	476.9 ± 210.8	4 (30.8)	4.7 ± 1.7	NA	1 (7.7)	0 (0)	NA
	**OLR (** *n* ** = 25)**	190.2 ± 51.8	628.0 ± 231.0	8 (32.0)	7.2 ± 2.3	NA	1 (4.0)	0 (0)	NA
	***P value***	***0.001***	***< 0.001***	*0.916*	***0.002***	*NA*	*0.890*	*0.999*	*NA*
Zhang et al. [44]	**RLR (** *n* ** = 7)**	225.43 ± 44.75	214.29 ± 265.70	NA	7.29 ± 4.31	0 (0)	3 (42.85)	0 (0)	7,305 (6,763-7,847)
	**OLR (** *n* ** = 15)**	235.67 ± 14.42	521.33 ± 246.34	NA	11 ± 4.96	—	6 (40)	0 (0)	4,190 (3,460-4,920)
	***P value***	*0.423*	***0.015***	NA	***< 0.001***	—	*0.079*	*0.999*	***< 0.001***
Zhang et al. [45]	**RLR (** *n* ** = 43)**	270 (120–480)	200 (50-1500)	3 (7)	7 (4–13)	2 (4.7)	5 (11.6)	0 (0)	NA
	**LLR (** *n* ** = 86)**	240 (120–480)	200 (50-2500)	14 (16.3)	7.5 (4–29)	9 (10.5)	20 (23.3)	0 (0)	NA
	***P value***	*0.062*	*0.418*	*0.141*	***0.016***	*0.265*	*0.115*	*0.999*	*NA*
Wu et al. [46]	**RLR (** *n* ** = 12)**	160 (140–180)	20 (16.2–27.5)	NA	4.5 (4–7)	0 (0)	5 (41.7)	0 (0)	NA
	**OLR (** *n* ** = 8)**	309 (172–394)	90 (50–550)	NA	9 (6.8–11.8)	—	1 (12.5)	0 (0)	NA
	***P value***	***0.049***	***0.001***	*NA*	***0.017***	*—*	*0.325*	*0.999*	*NA*

**Fig. 4:**
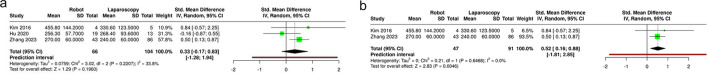
**a** Operative time: RLR vs LLR (all studies). **b **Operative time: RLR vs LLR (isolated liver resection)

*Operative Time* (Fig. 4a and b): Pooled across three comparative studies (66 robotic vs. 104 laparoscopic procedures), operative time did not differ significantly between the two approaches (SMD + 0.33, 95% CI − 0.17 to 0.83; *p* = 0.1960; I² = 33.8%; τ² = 0.0759), indicating broadly comparable operating times. When restricted to isolated hepatic resections (two studies; 138 patients: 47 robotic, 91 laparoscopic), the robotic approach was associated with a modestly but significantly longer operative time (SMD + 0.52, 95% CI 0.16 to 0.88; *p* = 0.0046; I² = 0%).

**Fig. 5:**
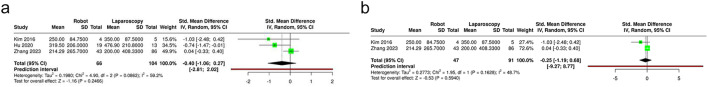
**a** Intraoperative blood loss: RLR vs LLR (all studies). **b **Intraoperative blood loss: RLR vs LLR (isolated liver resection)

*Intraoperative Blood Loss* (Fig. 5a and b): No significant difference in intraoperative blood loss was observed between robotic and laparoscopic resection (SMD − 0.40, 95% CI − 1.06 to 0.27; *p* = 0.2466; I² = 59.2%; τ² = 0.1980), with a non-significant trend favouring the robotic approach. The effect remained non-significant when restricted to isolated resections (SMD − 0.25, 95% CI − 1.19 to 0.68; *p* = 0.5940; I² = 48.7%; τ² = 0.2773).

**Fig. 6:**
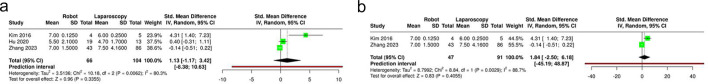
**a** Length of hospital stay: RLR vs LLR (all studies). **b **Length of hospital stay: RLR vs LLR (isolated liver resection)

*Length of Hospital Stay* (Fig. 6a and b): Length of hospital stay was comparable between approaches (SMD + 1.13, 95% CI − 1.17 to 3.42; *p* = 0.3355; I² = 80.3%; τ² = 3.5136). The result was unchanged in the isolated-resection subgroup (SMD + 1.84, 95% CI − 2.50 to 6.18; *p* = 0.4055; I² = 88.7%; τ² = 8.80).

### Robotic vs. open surgery: literature experiences

**Fig. 7:**
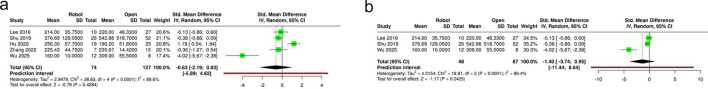
**a** Operative time: RLR vs OLR (all studies). **b **Operative time: RLR vs OLR (isolated liver resection)

*Operative Time* (Fig. 7a and b): Pooled across five comparative studies (74 robotic vs. 127 open procedures), operative time did not differ significantly between robotic and open resection (SMD − 0.63, 95% CI − 2.19 to 0.93; *p* = 0.4284; I² = 89.6%; τ² = 2.9479). The direction of effect favoured the robotic approach but did not reach significance and was characterised by very high heterogeneity. When restricted to isolated hepatic resections (three studies; 135 patients), the point estimate increased in magnitude while remaining non-significant (SMD − 1.40, 95% CI − 3.74 to 0.95; *p* = 0.2425; I² = 89.4%; τ² = 4.02).

**Fig. 8:**
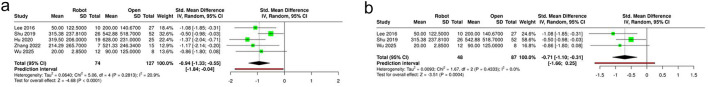
**a** Intraoperative blood loss: RLR vs OLR (all studies). **b **Intraoperative blood loss: RLR vs OLR (isolated liver resection)

*Intraoperative Blood Loss* (Fig. 8a and b): Robotic resection achieved significantly lower intraoperative blood loss than open surgery (SMD − 0.94, 95% CI − 1.33 to − 0.55; *p* < 0.0001; I² = 20.9%; τ² = 0.0640), with low heterogeneity and a consistent directional advantage across all five studies (robotic cohorts averaging 20–319 mL *versus* 90–628 mL for open cohorts). The advantage was confirmed in the isolated-resection subgroup, with negligible heterogeneity (SMD − 0.71, 95% CI − 1.10 to − 0.31; *p* = 0.0004; I² = 0%; τ² = 0.0093).

**Fig. 9:**
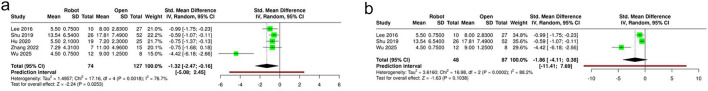
**a** Length of hospital stay: RLR vs OLR (all studies). **b **Length of hospital stay: RLR vs OLR (isolated liver resection)

*Length of Hospital Stay* (Fig. 9a and b): Robotic resection was associated with a significantly shorter hospital stay than open surgery (SMD − 1.32, 95% CI − 2.47 to − 0.16; *p* = 0.0253; I² = 76.7%; τ² = 1.4957); individual studies consistently favoured the robotic approach. When restricted to isolated resections, the effect retained a comparable magnitude but lost statistical significance (SMD − 1.86, 95% CI − 4.11 to 0.38; *p* = 0.1038; I² = 88.2%; τ² = 3.6160).

## Discussion

This study reports one of the largest single-surgeon consecutive series of RLR exclusively for benign hepatic diseases, encompassing a broad pathological and anatomical spectrum over a 12-year period. Moreover, we integrate our institutional experience with a PRISMA-compliant systematic review and meta-analysis.

The novelty of this work lies precisely in this exclusive focus: although several high-volume robotic series are available, they are almost invariably dominated by malignant indications or report benign cases only as small subgroups embedded within mixed cohorts. To our knowledge, no comparable consecutive series confined exclusively to benign disease, and no formal meta-analysis restricted to the benign setting, has previously been reported, which is the specific evidence gap the present study was designed to address.

Our findings show that RLR for benign disease demonstrates excellent safety and reproducibility: a 1.6% major complication rate (Clavien–Dindo ≥ III) with zero 90-day mortality and readmissions. A significant learning curve effect was documented—progressive reductions in operative time and hospital stay without increased morbidity. The IWATE score effectively stratified case complexity and predicted operative duration.

Compared with published LLR for benign diseases benchmarks, RLR achieves substantially lower conversion rates in posterosuperior segment resections [14, 47–49].

### Surgical issue

Surgical management of benign hepatic disease carries a distinctive ethical imperative: the decision to operate must always be weighed against the inherent risk of hepatectomy in patients who might otherwise live asymptomatic lives [50]. The proven advantages of minimally invasive liver surgery (reduced pain, shorter hospitalisation, faster return to daily function, and comparable or lower complication rates versus open surgery) are particularly relevant in this setting [23, 51–54]. In this regard, the use of three-dimensional reconstructions also provides a technical benefit to surgery, by enabling clearer and more precise preoperative planning [55–57].

The robotic platform extends these advantages into anatomically challenging areas that would otherwise necessitate OLR or incur prohibitive laparoscopic conversion rates. The 40.3% rate of lesions in posterosuperior difficult segments in our series reflects this deliberate extension of indications. In LLR, resection of segments I, IVa, VII, and VIII is associated with conversion rates up to 10%, longer operative times, and greater intraoperative blood loss compared with anterolateral resections [14, 58]. The wristed instruments, stable magnification, and ergonomic intuitiveness of the robotic system facilitate atraumatic dissection in these anatomical constraints, enabling precise parenchymal-sparing strategies that minimise unnecessary hepatic tissue sacrifice [59–62].

Our conversion rate of 3.2% was attributable to early-phase technical challenges (dense adhesions and haemostatic control), consistent with the expected learning curve rather than inherent platform limitations. This finding is clinically significant in the benign context, where avoidance of laparotomy maximises the minimally invasive benefit in patients without oncological urgency [63, 64].

The perioperative safety profile (no 90-day mortality, no readmissions, Clavien–Dindo ≥ III rate of 1.6%) compares favourably with published robotic series [65, 66]. The 13% major complication rate reported by Tsekouras et al. [18] in the most comprehensive systematic review of RLR for benign disease likely reflects the heterogeneity of case complexity and surgeon experience across pooled case series, whereas our results derive from a standardised single-surgeon programme. Furthermore, the integration of IOUS with ICG fluorescence represents a synergistic multimodal navigation approach as reported in many literature experiences [13, 67].

Combined colorectal–hepatic robotic procedures were performed in 16 patients (25.8%) without apparent increase in morbidity. This observation supports the feasibility of simultaneous robotic multi-organ surgery as previously reported by our group [68], and underscores the platform’s versatility in enabling complex integrated procedures within a single operative session, reducing the overall surgical burden on the patient.

### Learning curve

The learning curve analysis revealed statistically significant improvements in operative time (225 vs. 135 min; *p* = 0.033) and hospital stay (6 vs. 4 days; *p* = 0.004) from early to late experience, without any increase in morbidity or conversion rate. This trajectory is consistent with the current consensus suggesting that the learning curve for RLR is shorter than for LLR, particularly for surgeons with prior minimally invasive experience [69]. The comparable IWATE scores between phases (*p* = 0.299) confirm that performance improvements are attributable to a genuine learning effect rather than to progressive case simplification. This is further corroborated by the significant IWATE–operative time correlation in isolated hepatectomies (ρ = 0.60; *p* = 0.0004), validating the score’s predictive accuracy in the robotic context.

The trend toward longer Pringle times in the late phase (35 vs. 22.5 min; *p* = 0.074) warrants interpretation. Rather than indicating a technical regression, this likely reflects a deliberate evolution toward more demanding resections with higher vascular risk in the advanced experience period, a natural consequence of programme maturation, where greater technical mastery enables acceptance of cases requiring more prolonged hepatic pedicle control. The stable complication profile across both phases supports this interpretation.

The present series also contributes evidence that the robotic learning curve is achievable without substantial prior laparoscopic liver experience, a scenario increasingly common in centres where robotic platforms were introduced as the primary minimally invasive hepatic modality. This has implications for training programme design and credentialing in the emerging era of robotic surgery [70].

### Comparison with literature experiences

Direct head-to-head comparison between RLR and LLR remains methodologically challenging due to selection bias, heterogeneity in case complexity, and the absence of randomised controlled trial data. Nonetheless, the available comparative evidence, including multiple propensity-score matched studies [38, 45], consistently supports the following: comparable operative times at matched complexity levels [33, 34, 38, 44, 45], lower conversion rates for robotic posterosuperior hepatectomies [45], comparable or marginally lower intraoperative blood loss [33, 38, 41, 44, 46] and superimposable complication rates [33, 34, 38, 41, 44–46]. The results of the present series align with these comparative benchmarks and extend them specifically to the benign disease population, a subgroup previously underrepresented in comparative analyses.

Robotic and laparoscopic resection showed comparable intraoperative blood loss (SMD − 0.40, *p* = 0.2466), with a non-significant trend favouring the robotic platform, indicating equivalent haemostatic performance of the two minimally invasive approaches in the benign setting. Operative time was likewise comparable overall (SMD + 0.33, *p* = 0.1960), although a modestly longer robotic operative time emerged in isolated resections (SMD + 0.52, *p* = 0.0046), plausibly reflecting the additional docking and set-up time intrinsic to the robotic workflow. Hospital stay was equivalent between approaches (SMD + 1.13, *p* = 0.3355), confirming that minimally invasive perioperative recovery benefits translate similarly to both robotic and laparoscopic platforms.

Across the 16 studies identified in our systematic review, a consistent pattern emerges: the robotic platform confers haemostatic advantage and accelerates postoperative recovery versus open surgery, whilst achieving operative times broadly equivalent to laparoscopic approaches at matched complexity levels.

Comparison against open approaches demonstrated more decisive robotic advantages. Robotic resection achieved significantly lower intraoperative blood loss than open surgery (SMD − 0.94, *p* < 0.0001; I² = 20.9%), a robust estimate characterised by low heterogeneity and a consistent direction of effect across all five studies. Operative time, by contrast, did not differ significantly between the two approaches (SMD − 0.63, *p* = 0.4284), with a non-significant trend favouring the robotic platform.

Length of hospital stay was significantly shorter with the robotic approach (SMD − 1.32, *p* = 0.0253), with individual studies demonstrating reductions of 2–4 days; the effect retained a comparable magnitude in isolated resections although it lost significance in the smaller subgroup (SMD − 1.86, *p* = 0.1038). These findings collectively underscore the perioperative advantage of robotic minimally invasive technique over open hepatectomy in the benign disease management paradigm [33, 41, 44, 46].

Our conversion rate of 3.2% (both events confined to the early programme phase) is consistent with Shu et al. [38] and Zhang et al. [45] propensity-matched robotic series.

Our Clavien–Dindo ≥ III rate of 1.6% represents the lower boundary of the published range: comparable series report rates of 0% [33, 34], 5.3% [41], and 6.25% [43]. The zero 90-day mortality and readmission rate observed in our series aligns with the universal absence of perioperative mortality across all robotic arms in the retrieved comparative studies.

The heterogeneity observed across the pooled analyses (I² ranging from 0% to 89.6%) reflects genuine variation in surgeon experience, case selection, institutional protocols, and geographic healthcare context rather than methodological deficiency; notably, the most clinically relevant estimate — reduced blood loss versus open surgery — showed low heterogeneity (I² = 20.9%).

Collectively, these data position the present series within the published robotic benchmarks for benign hepatic surgery, while extending the evidence base to a consecutive single-surgeon cohort of a size and pathological breadth not previously reported in this specific indication.

### Technological advantages and broader implications for hepatobiliary surgery

Beyond the comparative perioperative metrics, the advantages intrinsic to the robotic platform deserve specific emphasis, since they account for the outcomes observed in our series and define the distinctive role of the technology in benign hepatic surgery. The integration of three-dimensional stereoscopic magnification with fully wristed instruments offering seven degrees of freedom, motion scaling, and physiological tremor filtration reconstitutes, within the closed abdomen, the dexterity and depth perception that rigid laparoscopic instrumentation inevitably forfeits [10, 12, 59]. This translates into controlled, near-bloodless parenchymal transection and into the fine peri-hilar and perivascular dissection demanded when lesions abut the major hepatic veins, the inferior vena cava, or the Glissonean pedicles [10, 12, 59].

Such capabilities are most consequential precisely in the posterosuperior segments (I, IVa, VII, VIII), where the geometry of the subphrenic space and the unfavourable angle of approach underlie the higher conversion rates, longer operative times, and greater blood loss historically reported for laparoscopic resection. pooled robotic-versus-laparoscopic evidence confirms a reproducible reduction in conversion for these difficult locations [58, 71, 72]. In our cohort, the 40% of resections involving difficult segments, with a conversion rate of only 3.2% and without perioperative mortality.

Furthermore, the conventional minor/major dichotomy poorly reflects true operative difficulty, and the IWATE complexity score offers a more objective framework for case selection and for matching procedural complexity to surgeon experiences [73].

A second distinguishing feature is the seamless incorporation of multimodal intraoperative navigation into a single console-controlled workflow [13]. Routine ICG, employed in 88.7% of our cases, enabled real-time tumour localisation, negative-staining delineation of Glissonean territories, and the sensitive detection of biliary leakage, while intraoperative ultrasound provided continuous anatomical guidance. The synergy of these modalities allows the resection plan to be refined dynamically rather than fixed preoperatively [13].

The stable, surgeon-controlled camera and the substantially improved ergonomics further sustain precision during prolonged or anatomically demanding operations and attenuate the operator fatigue that accumulates over complex laparoscopic hepatectomy [74]. This benefit is especially pertinent to combined surgery: one quarter of our patients underwent simultaneous robotic colorectal–hepatic resection without an apparent increase in morbidity, illustrating how the stability and reach of the platform permit multi-quadrant, multi-organ procedures to be completed safely within a single operative session and a single minimally invasive access [68].

The implications of these features extend well beyond benign disease. Contemporary evidence suggests that the robotic learning curve is shorter, more reproducible, and less dependent on extensive advanced-laparoscopic experience than that of laparoscopic liver surgery — a property that may democratise access to minimally invasive hepatectomy and that bears directly on the design of structured training and credentialing pathways [69, 70, 75, 76]. In this respect, benign hepatic resection, in which oncological urgency is absent and the minimisation of morbidity is the overriding objective, constitutes an ideal low-risk environment in which a surgical team can consolidate robotic proficiency before progressing to malignant and major anatomical resections, a graduated stepping-stone strategy increasingly adopted by maturing hepatobiliary programmes.

The principal and legitimate objection to robotic surgery in this context remains its incremental cost; this calculus, however, is evolving, as the entry of competing platforms, the abbreviation of operative and learning times with accruing experience, and the demonstrable reductions in blood loss, transfusion requirement, and length of stay relative to open surgery progressively offset the initial outlay [23, 51–54]. Finally, the telemanipulated console provides the natural substrate for emerging precision-surgery adjuncts whose integration is more readily achieved in a digital operative environment than in conventional surgery [77, 78]. Interpreted in this light, the present series should be regarded not merely as evidence of safety within a circumscribed indication, but as a reproducible template informing the wider, staged implementation of robotic hepatobiliary surgery.

### Limitations

The principal limitations merit careful consideration. This retrospective, single-centre investigation with a non-randomised, observational design is inherently susceptible to selection bias; the absence of a synchronous institutional laparoscopic control group limits the strength of comparative conclusions.

We acknowledge that the absolute number of patients is modest; however, surgical candidates with purely benign hepatic disease are comparatively uncommon, and the present cohort represents, to our knowledge, the largest benign-exclusive robotic experience reported to date, whereas the larger published robotic series are predominantly oncological or mixed in composition. It was precisely to mitigate the constraints of a single-centre sample that the parallel PRISMA-compliant meta-analysis was incorporated, broadening the evidence base beyond our institution and situating the institutional findings within a quantitatively pooled comparative context.

Furthermore, the broad pathological spectrum of benign hepatic disease, while reflecting the real-world case-mix of a referral programme, inevitably introduces variability that constrains the applicability of findings to individual disease subsets. This heterogeneity precluded both robust subgroup stratification and the application of CUSUM or RA-CUSUM modelling for learning-curve assessment. A formal cost-effectiveness analysis was likewise not feasible within the available dataset.

We acknowledge as a limitation the absence of prospective PROSPERO registration; the review was, however, conducted under a pre-specified protocol fully compliant with PRISMA 2020.

Systematic collection of health-related quality-of-life metrics and long-term disease-specific outcomes, including recurrence and reoperation rates, was not performed. These gaps underscore the need for future prospective, multicentre studies employing rigorous comparative methodologies (randomisation or propensity-matching), standardised patient-reported outcome measures, and formal cost-effectiveness analyses to elucidate the definitive role of robotic hepatectomy within the contemporary management paradigm of benign liver disease.

## Conclusions

RLR for benign hepatic diseases is safe, feasible, and associated with low major morbidity and zero perioperative mortality in an experienced single-surgeon programme. The learning curve is achievable within a structured institutional programme and is reliably characterised by the IWATE difficulty score, which significantly correlates with operative time in isolated robotic hepatectomies.

The present series contributes the largest consecutive single-surgeon dataset exclusively addressing benign indications and, together with the systematic review, supports the role of RLR as a valid minimally invasive modality for technically challenging benign hepatic resections. Furthermore, RLR for benign liver diseases could serve as an ideal model for optimizing the surgical team’s proficiency prior to implementing a broader oncological liver resection program.

Prospective multicentre studies addressing health-related quality of life and cost-effectiveness are required to optimise patient selection and training pathways.

## Data Availability

No datasets were generated or analysed during the current study.
